# A survey of workplace violence against physicians in the hospitals, Myanmar

**DOI:** 10.1186/s13104-018-3240-x

**Published:** 2018-02-15

**Authors:** Yuichi Kasai, Tetsutaro Mizuno, Toshihiko Sakakibara, Si Thu, Thein Aung Kyaw, Kyaw Aung Htun

**Affiliations:** 1 Department of Spinal Surgery and Medical Engineering, Mie University Graduate School of Medicine, 2-174 Edobashi, Tsu, Mie Prefecture 514-8507 Japan; 2 Department of Orthopaedic Surgery, University of Medicine, Mandalay, 30th Street, Between 73rd & 74th Streets, Mandalay, Myanmar; 3 Department of Orthopaedic Surgery, University of Medicine, Yangon-1, No. 245, Myoma Kyaung Street, Lanmadaw Township, Yangon, Myanmar; 4 Department of Orthopaedic Surgery, (1000) bedded Nay Pyi Taw General Hospital, Nay Pyi Taw, Myanmar

**Keywords:** Hospital, Least developed country, Physician, Workplace violence

## Abstract

**Objective:**

Workplace violence in hospitals is recently becoming a major global concern in many countries. However, in Myanmar, we have felt that patients and their families have rarely made unreasonable complaints in hospitals, and then, the purpose of this study is to report the current state of workplace violence in hospitals in Myanmar. Participants are 196 physicians (108 males and 88 females) in hospitals in Myanmar.

**Results:**

A descriptive survey was conducted in regard to verbal abuse and physical violence from patients or the people concerned. At the results of this study, the percentages of physicians who have encountered verbal abuse and those who have encountered physical violence are markedly low (8.7 and 1.0%, respectively). The present study is the first to report the frequencies of verbal abuse and physical violence against physicians in a least developed country, and the results of the present study are important in terms of discussing workplace violence in hospitals.

## Introduction

Healthcare providers, such as physicians and nurses, are often subject to verbal abuse and physical violence from patients and their families in hospitals [1–3]. In 2015, the authors [4] conducted an investigation involving 110 orthopaedists from Mie Prefecture in Japan regarding patients who often made unreasonable complaints. As a result, the number of physicians who had encountered verbal abuse from patients or the people concerned such as their families during the previous 1 year was very high [n = 71 (64.5%)], and 6 physicians (5.5%) had encountered physical violence from patients and/or their families during the previous 5 years. In the contemporary society, as the awareness of patients and their families regarding their medical rights has increased, so have the levels of their demands for healthcare providers. We assume that, in many cases, these escalated levels of their demands have led to unacceptable and unreasonable demands/complaints, resulting in verbal abuse and physical violence, and then, workplace violence in hospitals is becoming a major global concern. Among the developed countries the United Kingdom of Great Britain and northern Ireland [1], Australia [2], Italy [3] and United States of America [5] and developing countries (Palestine [6], Brazil [7], Barbados [8], Turkey [9], and China [10]), the incidence rates of verbal abuse and physical violence are reported as shown in Table 1. Although simple comparisons cannot be made due to differences in subjects, years in which surveys were conducted, and survey methods among these countries, it is considered that, in many countries, more than 50% of the healthcare providers suffer verbal abuse, and several to several tens percent of the healthcare providers suffer physical violence.

**Table 1:** Incidence rates of verbal abuse and physical violence in several countries

Author’s name	Published year [reference number]	Country	Verbal abuse (%)	Physical violence (%)
Ness et al.	2000 [1]	United Kingdom	54.0	6.0
Forrest et al.	2011 [2]	Australia	58.0	18.0
Terzoni et al.	2015 [3]	Italy	40.2	11.5
Behnam et al.	2001 [5]	USA	75.0	21.0
Kitaneh et al.	2012 [6]	Palestine	62.2	23.2
Da Silva et al.	2015 [7]	Brazil	52.5	2.3
Abed et al.	2016 [8]	Barbados	60.0	3.0
Erdur et al.	2015 [9]	Turkey	43.1	5.2
Shi et al.	2015 [10]	China	92.8	81.0

As the authors have been providing medical support and performing instructive surgeries in Myanmar since 2010, we have felt that patients and their families have rarely made unreasonable complaints in hospitals. In least developed countries designated by the United Nations including Myanmar, there have been no reports concerning verbal abuse or physical violence from patients and the people concerned in hospitals. Against this background, we report the result of our investigation regarding the current state of verbal abuse and physical violence from patients or the people concerned such as their families in hospitals in Myanmar.

## Main text

The study subjects were comprised of physicians working in hospitals in three major cities including Yangon City (population: about 7 millions), Mandalay City (population: about 2 millions), and Naypyidaw City (capital city, population: about 1 million) in Myanmar (population: 52 millions), because authors visited these hospitals to perform instructive surgeries in 2015. This study was designed as a descriptive survey, and subjects of the questionnaire were 196 medical doctors (108 males and 88 females) working at a public orthopaedic hospital or a private general hospital in Yangon, all the physicians working at a public general hospital in Nay Pyi Taw, and all the physicians working at a public orthopaedic hospital in Mandalay at the time of our visiting. The number of 196 subjects is equivalent to 0.6% of all Myanmar medical doctors (about 30,000 persons, reported by Ministry of Health, Myanmar, in 2015). The collection rate of the questionnaire was 28 (82.3%) of 34 subjects in an orthopaedic hospital in Yangon, 30 (83.3%) of 36 subjects in a private hospital in Yangon, 123 (81.5%) of 151 subjects in a general hospital of Nay Pyi Taw, 15 (88.2%) of 17 subjects in an orthopaedic hospital in Mandalay, thus achieving retrieval rates ≥ 80% in each hospital.

According to the segmentation based on age of all subjects, 94, 92, 7, and 3 subjects were in their 20s, 30s, 40s, and 50s, respectively. The numbers of specialists and doctors-in-training were 76 and 120, respectively. Furthermore, the numbers of physicians working in public hospitals and a private hospital were 166 and 30, respectively.

A survey was anonymously conducted to ask the following two questions in English: (1) “Have you encountered verbal abuse (psychological violence such as clearly unreasonable demands, blackmailing, threats, or sexual harassment) from patients or the people concerned such as their families during the previous 1 year?” (To be answered with a Yes or No response), if the answer is yes, what kind of verbal abuse have you encountered? And (2) “Have you encountered violence (physical violence such as hitting, kicking, or throwing objects) from patients or the people concerned during the previous 5 years?” (To be answered with a Yes or No response), if the answer is yes, what kind of violence have you encountered?

The answers to these questions were sorted according to the subjects’ sex (male or female), physicians’ status (specialists or doctors-in-training), and type of hospital (public or private) and the results of both variables were compared. For statistical analysis in regard to sex, physicians’ status or type of hospital, Pearson’s Chi square tests were performed using Microsoft Office Excel 2016 because of statistical tests for two independent valuables, and a p value of less than 0.05 was considered significant. This study was performed under approval (No. 1714) from the ethics committee of Mie University, Japan.

All results of this study are summarized in Table 2. Of the 196 Myanmar physicians, 17 (8.7%) reported that they had encountered verbal abuse from patients or the people concerned such as their families. The categories of verbal abuse were ‘threats’ in 15 physicians and ‘clearly unreasonable demands’ in 2 physicians. No significant differences (p = 0.747) were noted between the sexes [10/108 (9.2%) for males, and 7/88 (8.0%) for females]. In addition, no significant differences (p = 0.758) were noted between the specialists [6/76 (7.9%)] and the doctors-in-training [11/120 (9.2%)]. The percentage [7/30 (23.3%)] of physicians at the private hospital who had encountered verbal abuse from patients or the people concerned was significantly higher (p = 0.002, p < 0.01) than that at the public hospitals [10/166 (6.0%)].

**Table 2:** Verbal abuse and physical violence against physicians in the hospitals, Myanmar

	Verbal abuse (n = 17)	Statistical significance (p value)	Physical violence (n = 2)	Statistical significance (p value)
Male physician (n = 108) Female physician (n = 88)	10 (9.2%) 7 (8.0%)	ns (0.747)	1 (0.9%) 1 (1.1%)	ns (0.884)
Specialists (n = 76) Doctors-in training (n = 120)	6 (7.9%) 11 (9.2%)	ns (0.758)	1 (1.3%) 1 (0.8%)	ns (0.743)
Public hospital (n = 166) Private hospital (n = 30)	10 (6.0%) 7 (23.3%)	p < 0.01 (0.002)	1 (0.6%) 1 (3.3%)	ns (0.171)

The number of Myanmar physicians who had encountered physical violence from patients or the people concerned such as their families was very low [2/196 (1.0%)]. The category of physical violence was ‘throwing objects’ in 2 physicians. No significant differences were noted between the sexes [1/108 (0.9%) for males, and 1/88 (1.1%) for females] (p = 0.884). In addition, no significant differences were noted between the specialists [1/76 (1.3%)] and the doctors-in-training [1/120 (0.8%)] (p = 0.743), as well as between the public hospitals [1/166 (0.6%)] and the private hospital [1/30 (3.3%)] (p = 0.171).

In discussion, it is frequently reported that, due to verbal abuse and physical violence from patients or the people concerned such as their families in hospitals, healthcare providers such as physicians and nurses feel marked mental stress or suffer from physical injures and, as a result, they have no choice but to resign or take time off work [1–3]. This is one contributory factor for the collapse of the healthcare systems. However, in the present study in Myanmar, the percentages of physicians who have encountered verbal abuse and those who have encountered physical violence are markedly low (8.7 and 1.0%, respectively). The present study is the first to report the frequencies of verbal abuse and physical violence against physicians in a least developed country.

We have discussed the causes and background of in-hospital verbal abuse and physical violence. They can be attributed to insufficient explanations or inadequate approaches to patients/families on the part of healthcare providers, as well as by an abnormal personality, lack of knowledge, and overreactions, etc. on the part of patients’. However, the increasing awareness of patients of their medical rights in recent years, and their increasing distrust of medical institutions may have been major causes of such abuse and violence. We assume that, in Myanmar, verbal abuse and physical violence do not frequently occur because: patients have a low-level awareness of their medical rights as they can receive medical consultations for free or at a low cost, physicians are relatively respected by patients, people do not assert themselves frequently, people have the cultural perception that they should respect others, and people devotedly practice Buddhism in which they are taught the importance of non-violence. In addition, in the present study, when comparing public hospitals in which medical expenses are basically free with a private hospital in which patients have to pay all medical expenses (including service fees), the frequencies of verbal abuse and physical violence are relatively higher for the latter. On this basis, we assume that patients who go to private hospitals have a higher-level awareness of their medical rights because of the need to pay medical expenses. Authors have speculated that patients’ low-level awareness of their medical rights or religious devotion may be related to low frequency of verbal abuse and physical violence from patients and their families in hospitals.

## Limitations

The present study has had some limitations, examples of which are: (1) the number of subjects is very low (n = 196), and it accounts for only 0.6% of all physicians throughout Myanmar (approximately 30,000) and most subjects are in their 20s or 30s, and thus, there is a need to accumulate more subjects in order to better describe the country’s entire trend, (2) the present study has involved three public hospitals and one private hospital, and these numbers are too low to describe the country’s entire trend, (3) we have not thoroughly investigated the departments to which the subjects belong, or details of the verbal abuse/violence that have been reported, (4) the verbal abuse and physical violence from medical colleagues and supervisors were not included in this study, and (5) our data may not be simply compared with the incidence rates of verbal abuse and physical violence in other countries. We need further investigations in Myanmar, however, the present results are important, because authors have indicated the possibility that patients’ low-level awareness of their medical rights or religious devotion may decrease workplace violence in hospitals.
